# Manipulation of host response and trajectory of parasite survival: A damage-response framework perspective

**DOI:** 10.1080/21505594.2026.2732345

**Published:** 2026-09-14

**Authors:** Kritsada Pruksaphon, Nopawit Khamto, Artid Amsri, Maria de Lourdes Pereira, Joshua D. Nosanchuk, Sirida Youngchim

**Affiliations:** a Department of Medical Technology, School of Allied Health Sciences, Walailak University, Nakhon Si Thammarat, Thailand; b Center of Excellence Research for Melioidosis and Microorganisms (CERMM), Walailak University, Nakhon Si Thammarat, Thailand; c Department of Biochemistry, Faculty of Medical Science, Naresuan University, Phitsanulok, Thailand; d Office of Research Administration, Chiang Mai University, Chiang Mai, Thailand; e Department of Microbiology, Faculty of Medicine, Chiang Mai University, Chiang Mai, Thailand; f CICECO-Aveiro Institute of Materials and Department of Medical Sciences, University of Aveiro, Aveiro, Portugal; g Department of Medicine (Division of Infectious Diseases) and Department of Microbiology and Immunology, Albert Einstein College of Medicine, Bronx, NY, USA

**Keywords:** Damage-response framework (DRF), immunopathology, inflammation, parasitic infection, infectious diseases, infection, Colonizing Opportunistic Pathogens

## Abstract

The Damage-Response Framework (DRF) provides a conceptual model for understanding the pathogenesis of parasitic infections by distinguishing biological infection from clinical infectious disease. Rather than viewing parasite virulence as an intrinsic property, the DRF proposes that disease results from dynamic host–parasite interactions, with host damage determined by the magnitude and quality of the immune response. Both insufficient immune responses, permitting parasite proliferation, and excessive inflammation, causing immunopathology, can lead to disease. Using representative examples, including *Blastocystis* spp. *Toxoplasma gondii*, and *Trichinella spiralis*, this review illustrates how similar parasites produce diverse clinical manifestations depending on host immune status, parasite characteristics, and environmental factors. The DRF also highlights the importance of host damage as a unifying endpoint that explains disease heterogeneity across parasitic infections. Integrating this framework into parasitology provides a foundation for developing mechanism-based diagnostics, immunomodulatory therapies, and personalized intervention strategies while identifying priorities for future translational medicine.

## GRAPHICAL ABSTRACT

**Figure:**
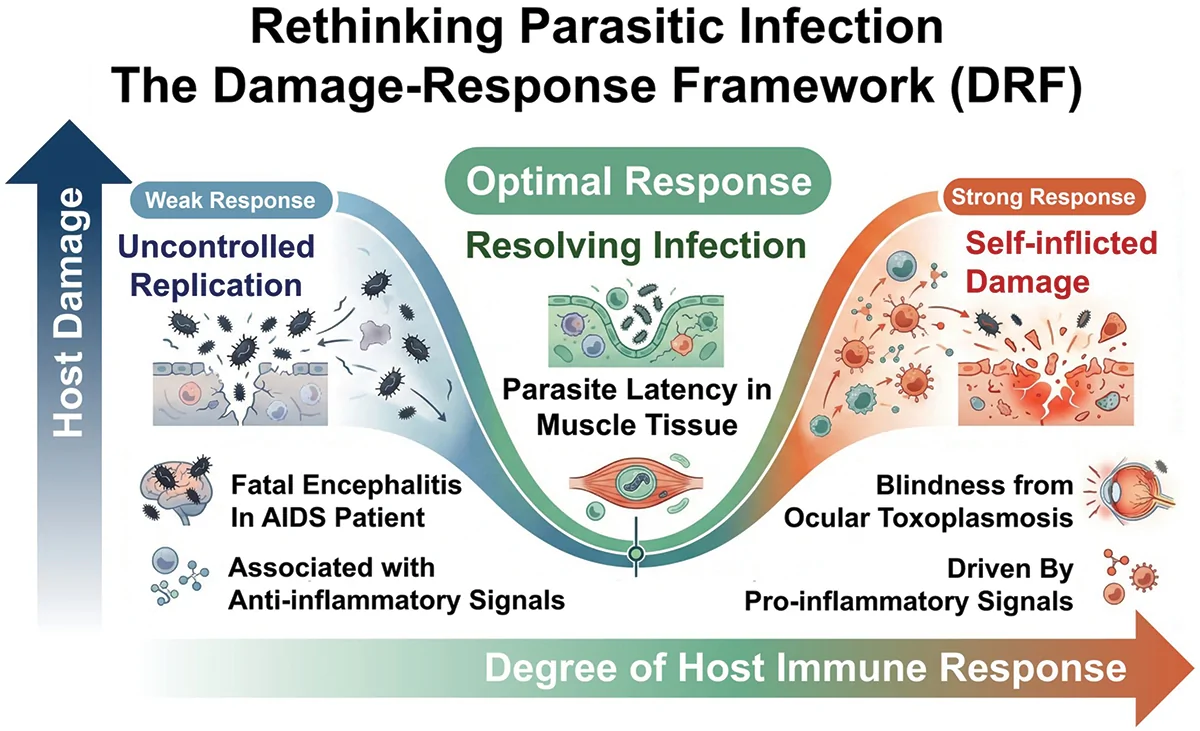

## Introduction: the critical distinction between “Infection” and “Infectious diseases”

*“Infection is common but disease is rare”* Arturo Casadevall. Immunity to invasive fungal diseases. Annu Rev Immunol. (2022) [1,2]

The statement cited above prompts critical reflection and thoughtful consideration of its potential implications within the field of infectious diseases. At its most fundamental, an infection is a biological process defined by a host’s acquisition of a disease-causing agent, or pathogen. Definitions and terminology related to the acquisition of microorganisms by humans and animals have been widely adopted in the scientific literature. Acquisition refers to the successful entry of a microorganism into the host through routes such as inhalation, ingestion, direct contact, or vector-mediated transmission. However, acquisition alone does not necessarily result in infection. Contamination, in contrast, describes the mere presence of microorganisms on body surfaces, clinical specimens, or medical devices without microbial establishment, sustained replication, tissue invasion, or induction of host responses. In fact, with nearly every breath, we encounter potentially problematic microbes. As Dr. Casadevall notes, most such acquisitions of a microbe are infections that do not result in disease as the host is rapidly able to block the pathogen by effective innate and/or adaptive responses. Disease occurs when the encountered microbe successfully breaches a host’s initial defenses and establishes a foothold. The term “pathogen” encompasses a vast and diverse array of agents, including bacteria, viruses, fungi, and parasites, each with unique mechanisms for entering and replicating within a host. For example, *Mycobacterium tuberculosis* can establish in the lungs and disseminate, while respiratory viruses like SARS-CoV-2 primarily target specific pulmonary cells [3–5]. Fungi like Candida can cause superficial or systemic infections [6,7], and parasites such as the protozoan *Plasmodium* spp. are the causative agents of malaria that can cause subclinical to lethal infections.

The process of infection can be conceptualized as a sequence of analogous events to a “Trojan horse” hypothesis [8,9]. The initial entry of the pathogen into the host is the first step. Once inside, the pathogen must survive the host immediate environment and initiate acquisition or replication. More precisely, pathogens must establish themselves within the host and, in many cases, undergo replication to maintain a biologically significant presence. Although microbial replication is an important feature of infection, it should not be regarded as the sole criterion distinguishing infection from contamination. Rather, infection is characterized by microbial persistence within host tissues together with biologically meaningful interactions with host defense mechanisms. This replication is the key feature that distinguishes infection from mere exposure or transient contamination. The ease with which a pathogen can successfully complete these steps, entry, survival, and multiplication, is a measure of its infectivity [10]. Infectivity refers to the ability of a microorganism to establish infection within a susceptible host. Consequently, highly infective agents are capable of initiating infection even after exposure to a relatively small initial inoculum, which is defined as the number of microorganisms encountered by the host at the onset of exposure. Importantly, infection is defined by this microbiological activity and the host interaction to them, regardless of whether the host becomes ill. The host immune system recognizes the invading pathogen as foreign and mounts a response, leading to a dynamic interaction between the microbe and host. Although this interaction is sometimes described metaphorically as a biological conflict, it is more accurately viewed as a dynamic interplay between pathogen-associated factors and host defense mechanisms that ultimately determines the extent of host damage and clinical outcome. This interaction, this biological conflict, is the essence of infection. The process can be localized to a specific tissue or become systemic spreading throughout the body, but it remains fundamentally a microbiological event that may or may not proceed to a state of clinical illness. Importantly, microbial replication or dissemination does not inevitably result in symptomatic disease. Many infections remain asymptomatic or subclinical despite evidence of pathogen persistence and host immune recognition, highlighting that infection and disease represent distinct biological concepts [11].

In contrast to infection, an infectious disease represents the clinical outcome of infection and is characterized by a measurable disruption of normal physiological functions. It is the state of being ill, marked by the presence of signs and symptoms. However, infectious diseases encompass a broad spectrum ranging from asymptomatic and subclinical infections to severe life-threatening illnesses. Therefore, the presence of infection does not necessarily imply overt clinical manifestations, and disease severity reflects the degree of host damage rather than the mere presence of microorganisms. When germs invade and multiply, the process can cause disease that leads to illness. This progression is not guaranteed; disease arises only when the ongoing battle between the pathogen and the host immune system leads to significant and persistent damage. This damage is not solely the result of the pathogen direct actions. While many pathogens produce various virulence factors, such as toxins or proteolytic enzymes, that directly harm host cells, a substantial portion of the pathology associated with infectious disease is iatrogenic, caused by the immune response [12]. In this context, iatrogenic damage refers broadly to host injury resulting from medical interventions or therapeutic manipulation of the immune system that inadvertently contributes to tissue damage or alters disease outcomes. The immune system, in its effort to eliminate the foreign invader, can cause significant incidental damage to surrounding tissues. The cardinal signs of a localized microbial infection – redness (rubor), heat (calor), swelling (tumor), and pain (dolor) – are the direct result of the inflammatory process orchestrated by the innate immune system to combat the any pathogens [13].

The symptoms of a disease are the tangible, clinical expression of this underlying conflict. They can be general, affecting the whole body, such as fever, fatigue, muscle aches, and loss of appetite, or they can be specific to the site of infection, such as a skin rash, coughing, or diarrhea [14]. Example of fever, a pathogen does not produce the fever, rather fever is a host response triggered by the immune system recognizing microbial factors and the elevation in temperature is an attempt by the host to render the environment less favorable for microbial proliferation and survival. Therefore, disease represents a state where the host-pathogen interaction has tipped out of balance, resulting in physiological or pathological changes and clinically apparent illness. The causal chain is clear, exposure to a pathogen may lead to infection, which in turn triggers an immune response. If this response fails to quickly and efficiently clear the pathogen, or if the response itself is overly aggressive, the resulting host response and/or damage manifests as disease [15]. Therefore, for this reason, the principle “*infection is common, disease is rare*” arises from the fact that this chain of events is frequently interrupted before appreciable host damage occurs. The following sections of this article will examine in detail how and why this interruption occurs, with a particular focus on parasitic infection and disease.

In this context, which provides the basis for the following section, the present review aims to synthesize the DRF as a unifying conceptual framework for understanding the pathogenesis of parasitic diseases. Specifically, the comprehensive review first examines the biological basis of host–parasite reciprocal interactions, highlighting how parasite virulence, immune evasion strategies, and host immune responses collectively determine the balance between asymptomatic infection and clinical disease. It then explores the fundamental principles of the DRF, including the dynamic relationship between host immune responses and host damage, and discusses how this framework redefines disease as an emergent property of host–parasite interactions rather than a fixed consequence of parasite virulence alone. Finally, representative parasitic diseases are presented to illustrate the applicability of the DRF across diverse host–parasite systems and to demonstrate its value in explaining clinical heterogeneity, disease progression, and the implications for host-directed therapeutic strategies and future research in parasitology.

## The biology of the host-parasite reciprocal interaction

To fully understand the Damage-Response Framework (DRF), it is essential to begin with an intensive analysis of the complex biological interactions that govern parasitic infections. The clinical manifestations of all parasitic diseases represent the net outcome of a dynamic interplay in which both the invading pathogens and the defensive responses contribute to tissue damage [16]. This section systematically deconstructs these mechanisms to establish the conceptual foundations of damage that the DRF is designed to model and explain in a structured manner.

### Direct pathogenesis: damage mediated by the parasite

The first source of damage in a parasitic infection is the direct physical and biochemical harm inflicted by the parasite itself as it invades, migrates, feeds, and/or reproduces within the host. Within the DRF, direct pathogenesis refers to host damage that arises primarily from parasite activities rather than from dysregulated host immune responses. This form of damage can be broadly categorized into physical damage, resulting from the mechanical presence or movement of parasites within host tissues, and biochemical damage, which is mediated by parasite-derived molecules or metabolic activities that directly disrupt host cellular functions and tissue integrity. These actions represent the “microbial factor” or “virulence factor” component of damage within the DRF and are often most prominent in acute or high-burden infections. These examples demonstrate that direct parasite-induced injury represents one component of the broader host–parasite reciprocal interaction described by the DRF, where biological damage reflects the combined effects of parasite replication and host immunity [17].

Parasites, by definition, are dependent on their host for survival and propagation, and this dependency manifests as a form of consumer–resource interactions [18]. They compete with the host for essential nutrients, absorbing vitamins, minerals, and biological macromolecules directly from the host diet or tissues. This process represents a form of direct resource sequestration, whereby parasites acquire or deplete essential host nutrients – including iron, hemoglobin, glucose, lipids, amino acids, and micronutrients – for their own growth and reproduction. Such nutrient competition may impair normal host physiology even in the absence of marked tissue destruction [19]. In chronic infections, especially in nutritionally compromised individuals, this constant drain of resources can lead to significant pathology, including malnutrition, iron-deficiency anemia, and stunted growth in children [20].

The main mechanism of biochemical direct damage is enzymatic tissue destruction. Many protozoan and helminthic parasites secrete a potent arsenal of lytic and proteolytic enzymes, which are essential for their life cycle. These enzymes degrade components of the host extracellular matrix, disrupt the integrity of mucosal epithelia, and facilitate parasite invasion of tissues and migration to its preferred niche. These enzymatic activities not only pave a structural route for parasite migration but also induce cellular death and tissue injury [21]. Beyond proteolytic enzymes, parasite derived excretory–secretory (ES) products, pore-forming proteins, phospholipases, hemolysins, and toxic metabolic by-products may directly alter cellular homeostasis, promote apoptosis or necrosis, and compromise organ function. Overall, these parasite-derived molecules constitute the principal biochemical mechanisms of direct pathogenesis. In contrast, physical damage results from the mechanical effects of parasite burden, migration, occupation of anatomical spaces, or repeated disruption of host cells. In addition to these biological mechanisms, the substantial physical presence of parasites, notably large multicellular helminths, may provoke serious pathology by causing blockage and exerting pressure on surrounding structures. In lymphatic filariasis, for instance, adult worms of species like *Wuchereria bancrofti*, *Brugia malayi*, and *Brugia timori* reside in lymphatic vessels. Their physical presence, combined with the host inflammatory response, can occlude the flow of lymph, leading to fluid accumulation, chronic swelling (lymphedema), and the disfiguring condition known as elephantiasis [22]. Similarly, heavy intestinal worm burdens can cause blockages, while parasites lodging in small-diameter venules can obstruct blood flow, leading to localized ischemia and organ dysfunction [23].

Moreover, many protozoan parasites cause damage through the direct hijacking and destruction of host cells. The *Plasmodium* species that cause malaria provide a classic example. During the human blood stage of their life cycle, these parasites invade erythrocytes, replicate asexually within them, and ultimately rupture the host cell to release a new generation of merozoites. This cyclical process of invasion and destruction is a direct cause of the profound anemia and associated pathologies frequently seen in malaria patients. The loss of red blood cells compromises oxygen delivery to tissues, contributing to the systemic organ dysfunction that characterizes severe parasitic disease [24–26]. Similar mechanisms are observed in several intracellular protozoa such as *T. gondii*, in which repeated cycles of host-cell invasion, intracellular replication, and cell lysis contribute directly to cumulative tissue damage independent of immune-mediated pathology [27].

### Indirect pathogenesis: immune mediated pathogenesis

While direct parasite-mediated damage can be significant, a central theme in modern parasitology is the recognition that in many chronic infections, the host immune response is the principal driver of pathology and clinical disease. The immune system, in its effort to control or eliminate the parasite, can trigger potent inflammatory and cell-destructive responses that inflict widespread secondary injury on host tissues. This host-mediated damage is the second critical component of the DRF and often accounts for the most severe and long-lasting consequences of parasitic disease [28,29]. Importantly, indirect pathogenesis encompasses a broad spectrum of host-mediated biological damage that extends beyond inflammation alone. It includes inflammatory responses, hypersensitivity reactions, immune complex-mediated injury, oxidative tissue damage, dysregulated immunoregulatory pathways, and chronic tissue remodeling or fibrosis [30]. Although these processes are interconnected, they differ substantially in their initiating stimuli, cellular mediators, molecular mechanisms, and pathological outcomes. Therefore, they should not be regarded as merely different manifestations of a single inflammatory process.

The key process of this indirect pathogenesis is inflammation. The host immune system recognizes parasitic antigens as foreign and initiates a defensive response, which is inherently inflammation [31]. This process involves the recruitment of immune cells like macrophages, neutrophils, eosinophils, and lymphocytes to the site of infection, and the release of a cascade of signaling molecules, including cytokines and chemokines. Depending on the infecting parasite and the immune microenvironment, these responses may be dominated by distinct immune pathways, including T_H_1, T_H_2, T_H_17, or regulatory T cell (T_Reg_)-associated responses, each producing unique biological consequences. While some inflammatory responses primarily function to eliminate parasites, others are characterized by allergic or hypersensitivity mechanisms, such as IgE-mediated mast cell activation and eosinophil degranulation, which represent immunopathological processes that are mechanistically distinct from protective anti-parasitic immunity [32]. While this inflammatory milieu is essential for containing the parasite, when the infection becomes chronic, the persistent inflammation leads to continuous, low-grade tissue damage, fibrosis, and eventual organ dysfunction. Furthermore, chronic infections frequently induce immunoregulatory pathways involving cytokines such as IL-10 and transforming growth factor-β (TGF-β), which limit excessive inflammation and prevent catastrophic tissue injury. However, prolonged immunoregulation may simultaneously promote parasite persistence, alter tissue homeostasis, and contribute to progressive pathological remodeling, illustrating the delicate balance between host protection and disease progression.

A well-established example of protective inflammation turning pathogenic is granuloma formation, the hallmark of schistosomiasis and intracellular protozoa [31,33]. Adult male and female schistosome flatworms live in the bloodstream, where they release large numbers of eggs. Many of these eggs become trapped in host tissues, particularly the liver or urinary bladder. In response, the host immune system mounts a vigorous inflammatory response, forming an organized structure “the granuloma” around each parasitic egg. This reaction serves a crucial protective function by compartmentalizing the highly antigenic and toxic substances released by the egg, thereby shielding the surrounding liver cells from direct damage [33]. However, the long-term consequences of this protective strategy are considerable. The chronic inflammation associated with granuloma formation triggers the activation of fibroblasts and the excessive deposition of collagen, leading to progressive liver fibrosis. This transition from protective immunity to pathological tissue remodeling exemplifies how persistent immune activation can shift from parasite containment toward irreversible organ damage. Thus, fibrosis represents not simply a consequence of inflammation but the culmination of aberrant wound-healing processes coordinated by immune cells, fibroblasts, and pro-fibrotic mediators, including TGF-β. Throughout the years, this scarring process can obstruct blood flow through the liver, causing portal hypertension and eventual liver failure. The granuloma is thus a perfect illustration of the immune response as a double-edged sword. Beyond schistosomiasis, immune-mediated pathology is also a defining feature of numerous other parasitic infections. In cerebral malaria caused by *P. falciparum*, excessive production of pro-inflammatory cytokines, together with endothelial activation and sequestration of parasitized erythrocytes, contributes to blood-brain barrier disruption and severe neurological manifestations [34,35]. Likewise, infected tissues exhibited significantly increased expression of pro-inflammatory cytokines accompanied by mild mononuclear inflammatory cell infiltration, indicating an active host immune response. These findings characterize the immunopathology of *Sarcocystis fusiformis* infection and provide insights that distinguish it from other *Sarcocystis* species, while highlighting the need for further studies on disease control and pathogenesis [36]. These examples collectively illustrate that clinical disease often reflects an imbalance in host immune responses rather than parasite burden alone, consistent with the central premise of the DRF.

An equally important aspect of host-parasite interactions is the development of immune regulatory mechanisms that limit excessive tissue damage but may inadvertently promote chronic infection. Persistent antigenic stimulation during long-lasting parasitic infections can induce immune exhaustion, a state characterized by diminished effector T-cell function, sustained expression of inhibitory receptors such as PD-1, CTLA-4, and TIM-3, reduced cytokine production, and impaired proliferative capacity [37]. Although immune exhaustion protects the host from uncontrolled immunopathology, it may simultaneously compromise parasite clearance and facilitate long-term persistence. In parallel, many parasites actively promote immune tolerance through expansion of regulatory T cells, alternatively activated macrophages (M2), and increased production of immunosuppressive cytokines such as IL-10 and TGF-β. Helminths provide particularly striking examples of this strategy, as they actively remodel the host immune environment toward a regulatory phenotype that suppresses excessive inflammation while enabling prolonged survival within the host. Similar regulatory pathways have also been reported in chronic protozoan infections, including leishmaniasis and toxoplasmosis, where IL-10 dependent immune suppression limits immunopathology but may also impair complete parasite elimination [38]. Within the framework of the DRF, these regulatory processes highlight the dynamic balance between protective immunity and immune-mediated damage. Insufficient immune activation results in uncontrolled parasite replication, whereas excessive inflammation causes collateral host tissue injury.

Another major mechanism of host-mediated damage is redox imbalance. Rather than representing an independent pathological process, oxidative stress is intimately integrated with the host inflammatory response and constitutes an essential component of innate immune defense [39]. As part of the anti-parasitic response, phagocytic cells like macrophages produce reactive oxygen species (ROS), in an attempt to kill the invading organism. However, when this response becomes amplified or persists, it exceeds the capacity of endogenous antioxidant systems, thereby precipitating a state of systemic oxidative stress. Reactive oxygen and nitrogen species (ROS and RNS) generated by activated macrophages, neutrophils, and eosinophils contribute not only to parasite killing but also to collateral injury of surrounding host tissues. Consequently, oxidative stress and inflammation act synergistically, creating a self-amplifying cycle in which inflammatory mediators stimulate oxidant production, while oxidative damage further perpetuates inflammatory signaling [40]. These highly reactive molecules do not discriminate between parasite and host; they damage host cell membranes, proteins and DNA, contributing significantly to the pathology of diseases such as cholangiocarcinoma (bile duct carcinoma) associated with chronic infections by *Opisthorchis viverrini* and *Clonorchis sinensis*, where both host- and parasite-derived factors exacerbate the oxidative burden and can lead to fatal malignancy [40–42].

Moreover, chronic parasitic infections are often characterized by a high and sustained load of circulating parasite antigens such as filariasis and schistosoma infections [43]. This can lead to the formation of large quantities of antigen-antibody complexes. While normally cleared by the immune system, these complexes can be deposited in small blood vessels, particularly in the kidneys and joints. This deposition can trigger the activation of the complement system (i.e. Arthus reaction), a powerful inflammatory cascade, leading to classic immunopathology that resembles systemic autoimmune diseases. Unlike protective inflammatory responses directed toward parasite elimination, immune complex-mediated diseases represent a distinct form of adaptive immunopathology in which host tissues become unintended targets of immune effector mechanisms. The resulting vasculitis and glomerulonephritis are manifestations of tissue damage caused not by the parasite itself, but by the host’s immune response to the infection [44,45].

### Persistence: parasite-mediated immunomodulation and escape

The capacity of numerous parasites, particularly helminths, to maintain long-term infections lasting years or even decades in an immunocompetent host demonstrates their highly evolved co-adaptive strategies [46,47]. Importantly, this capacity is not exclusive to helminths but is also characteristic of several protozoan parasites, including *T. gondii*, *Leishmania* spp., and other Apicomplexan organisms, which establish persistent infections through sophisticated modulation of host immune responses [48,49]. Parasites are not passive targets but skilled manipulators of the host immune system, shaping it in ways that promote their own survival. They employ a vast array of strategies to evade detection, suppress effective immune responses, and modulate the inflammatory environment to create a more favorable habitat [50,51]. Although the terms immune evasion and immune modulation are often used interchangeably, they represent distinct but complementary strategies employed by parasites to promote survival within the host. Thus, immune evasion enables parasites to escape immune attack, whereas immune modulation actively reshapes host immunity to establish a more permissive environment for chronic infection. In practice, these two strategies frequently coexist and act synergistically during the course of parasitic infections [52]. These mechanisms are crucial for understanding the chronicity of parasitic diseases and, critically, they directly influence the balance between parasite- and host-mediated damage [20,53]. However, parasite-induced immunomodulation should not be regarded as a uniformly beneficial or homeostatic process. Rather, it represents a dynamic and context-dependent phenomenon whose consequences depend on multiple factors, including parasite burden, tissue tropism, infection stage, and the immunological status of the host. Depending on these conditions, immune modulation may simultaneously restrict excessive inflammation, facilitate parasite persistence, or directly contribute to chronic tissue injury and fibrosis.

One of the most well-known evasion tactics is antigenic variation, employed by protozoa such as *Plasmodium* spp. and African trypanosomes. These parasites possess a large repertoire of genes encoding surface proteins and can periodically switch which gene is expressed, effectively changing their antigenic coat. This strategy allows a fraction of the parasite population to outpace adaptive immunity, which is confined to targeting antigenic variants that are no longer expressed [54,55]. Certain parasites engage in antigenic mimicry, incorporating molecular motifs that mirror host antigens, a strategy that attenuates immune visibility by promoting their classification as self-antigen [56].

**Figure 1.**
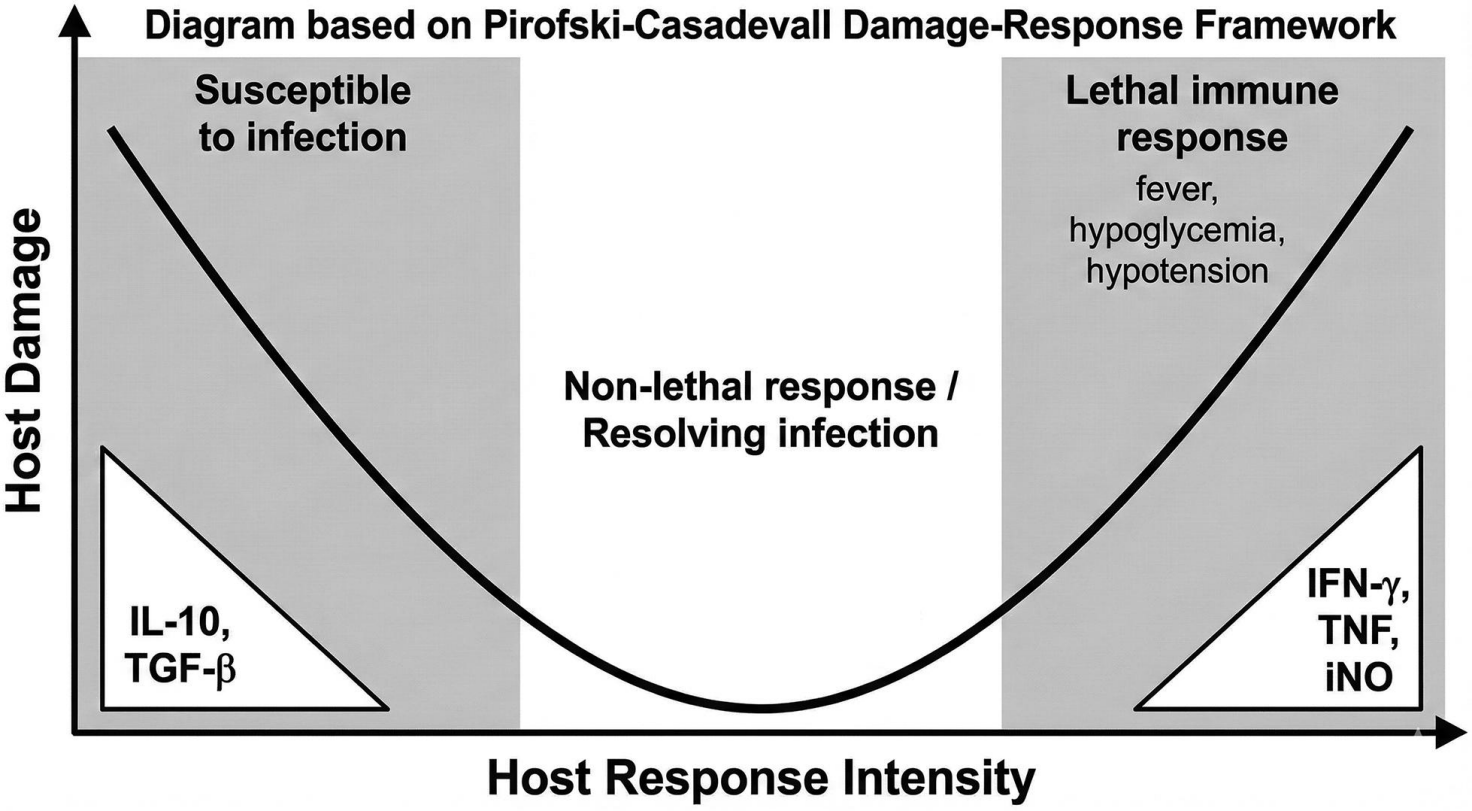
The profile of host cytokine response and the Pirofski-Casadevall DRF. This diagram illustrates the parabolic relationship between host response intensity and host damage. The framework demonstrates that host damage (disease) can occur at both extremes of the immune response spectrum. On the left, an insufficient or weak immune response leads to high damage due to unchecked microbial growth and susceptibility to infection, a state associated with anti-inflammatory mediators such as IL-10 and TGF-β. Conversely, on the right, an excessive or hyper-inflammatory response results in high damage caused by the immune system itself (immunopathogenesis), manifesting as fever, hypoglycemia, and hypotension. This severe response, which may be sufficiently intense to result in host mortality, is driven by pro-inflammatory factors such as IFN-γ, TNF-α, and inducible nitric oxide (iNO). The optimal outcome is found at the center (the trough of the curve), representing an intermediate response intensity where a balanced cytokine profile facilitates the resolution of infection with minimal damage to the host. Importantly, parasites themselves can engage with the host immune system in both directions, and either trajectory can lead to disease, manifesting as either acute or chronic illness (modified in part from Wynn & Kwiatkowski, [45]).

Perhaps the most profound strategy, particularly for helminths, is the active modulation of the host T-helper cell response. A robust, pro-inflammatory T_H_1-type response, characterized by cytokines like IFN-γ, is generally effective at killing intracellular pathogens but can also cause severe immunopathogenesis. Helminths have evolved mechanisms to actively suppress this T_H_1 response. Instead, they promote the development of T_H_2 and T_Reg_ cell responses. The T_H_2 response, characterized by cytokines like IL-4 and IL-13, is involved in tissue repair and is less effective at clearing the worms. T_Reg_ cells produce anti-inflammatory cytokines like IL-10 and TGF-β, which suppress the broader immune reaction. Nevertheless, this conceptual description should not be interpreted as implying that host immunity simply shifts along a single quantitative continuum from weak to strong responses. Contemporary immunology demonstrates that parasite persistence is determined by multiple interacting dimensions of the immune response, including its qualitative polarization, temporal dynamics, tissue-specific immune microenvironments, and complex regulatory networks involving regulatory lymphocytes, alternatively activated macrophages, and cytokine feedback loops. These mechanisms collectively shape disease progression and tissue pathology beyond the simplified representation illustrated in Figure 1 This altered immune microenvironment is highly beneficial to the parasite, as it prevents its elimination. Concurrently, it is also beneficial to the host, as it limits the incidental tissue damage that would result from a persistent, robust T_H_1 response, thereby promoting a tolerogenic immune environment [57–60]. This modulation is achieved through the secretion of a diverse array of immunomodulatory molecules. Parasites release ES products that can directly interfere with host immune signaling pathways, inhibit the activation and function of macrophages and induce apoptosis in dendritic cells. These molecules create a localized, and sometimes systemic, immunosuppressive environment that facilitates long-term parasite survival [61–63].

A traditional framework views these processes as separate, assuming that the parasite causes direct damage, the host produces additional harm through its response, and immune manipulation functions merely as a strategy that supports parasite survival. More modern perspectives, however, emphasize that these elements are tightly interwoven. Manipulation of immune response does far more than allow the parasite to escape detection; it actively shapes both the character and the intensity of host-driven pathogenesis. Accordingly, although the original DRF provides a valuable conceptual model by relating disease severity to the magnitude of host immune responses, it necessarily simplifies the biological complexity of host–parasite interactions. Indeed, tissue damage emerges from the integration of immune response magnitude with qualitative immune programs, spatial and tissue-specific organization, temporal progression, and regulatory immune circuits that continuously evolve throughout infection. By damping a potentially fatal T_H_1 response and favoring T_H_2/T_Reg_ activity, the organism in effect steers the immune system toward a particular pattern of tissue injury. This strategy avoids the threat of rapid, destructive inflammation, which would be lethal for both host and parasite, and instead results in a chronic, fibrotic condition that enables their long-term coexistence. The result is a tenuous, semi-commensal state in which parasite and host can co-exist. Ultimately, this interplay can be understood as the product of an evolutionary balancing act, in which both partners influence the nature and extent of damage. In this aspect, the clinical outcome of infection is not predetermined by either host or pathogen alone but emerges from their continuing biological interaction, a view that sits at the core of the DRF.

## The Pirofski-Casadevall DRF framework: deconstructing the interaction-based theory of parasite mediated pathogenesis

Throughout the past century, the study of infectious diseases has been dominated by two prevailing, yet incomplete, paradigms. The microbe-centric view, born from the foundational work of Robert Koch, conceptualized virulence as an intrinsic and stable property of a microorganism, determined by the presence of specific virulence factors. Conversely, the host-centric view attributed disease primarily to failure or deficiency in host defense systems. However, the clinical and scientific landscape of the late 20^th^ century presented profound challenges that these singular models could not adequately explain. The HIV/AIDS pandemic, in particular, brought this crisis into sharp focus, revealing that microbes previously considered harmless commensals, such as *Pneumocystis* (*carinii*) *jirovecii* [64] and *Blastocystis* spp [65], could become lethal pathogens in the context of a compromised immune system. This clinical reality demonstrated unequivocally that the same microbe could exist as a benign commensal or a deadly pathogen, with the outcome depending entirely on the host immune status. This invalidated the notion of virulence as a fixed parasitic trait [66].

The paradigm shift was further supported by advances in immunological theory. In particular, Polly Matzinger’s “The danger model” proposed that the immune system is driven not primarily by the ability to distinguish “self” from “non-self,” but by its capacity to detect damage or danger-associated signals released from stressed or injured tissues [67,68]. This shift from recognizing foreign entities to detecting harm opened the way for a new theory of pathogenesis, one that explains the dynamic interactions among the microbe, the host, and the damage arising from their engagement.

To resolve these paradoxes, Liise-anne Pirofski and Arturo Casadevall established the DRF, a contemporary principle first proposed in 1999 [69,70]. The framework was derived from the practical need to explain complex clinical observations and to instruct microbial pathogenesis in a way that reconciled the growing list of exceptions to classical models. The DRF is grounded on three fundamental and largely incontrovertible principles, which collectively redefine the nature of infectious disease including.

- Microbial pathogenesis is not an intrinsic property of either the microbe or the host alone; rather, it is an outcome of the interaction between the two. This principle shifts the analytical focus from the static attributes of the participants toward the dynamic nature of their encounter.
- The common denominator and critical outcome of the host-microbe interaction is the amount of host damage sustained. Disease becomes clinically apparent when this damage exceeds a certain threshold, disrupting physiological homeostasis.
- Host damage can arise from direct microbial factors (e.g. toxins, enzymes, tissue destruction caused by replication and space occupation) and/or from the host immune response. This phenomenon is crucial as it formally recognizes immunopathology, inflammatory damage, as a primary driver of disease, rather than just a side effect.

**Figure 2.**
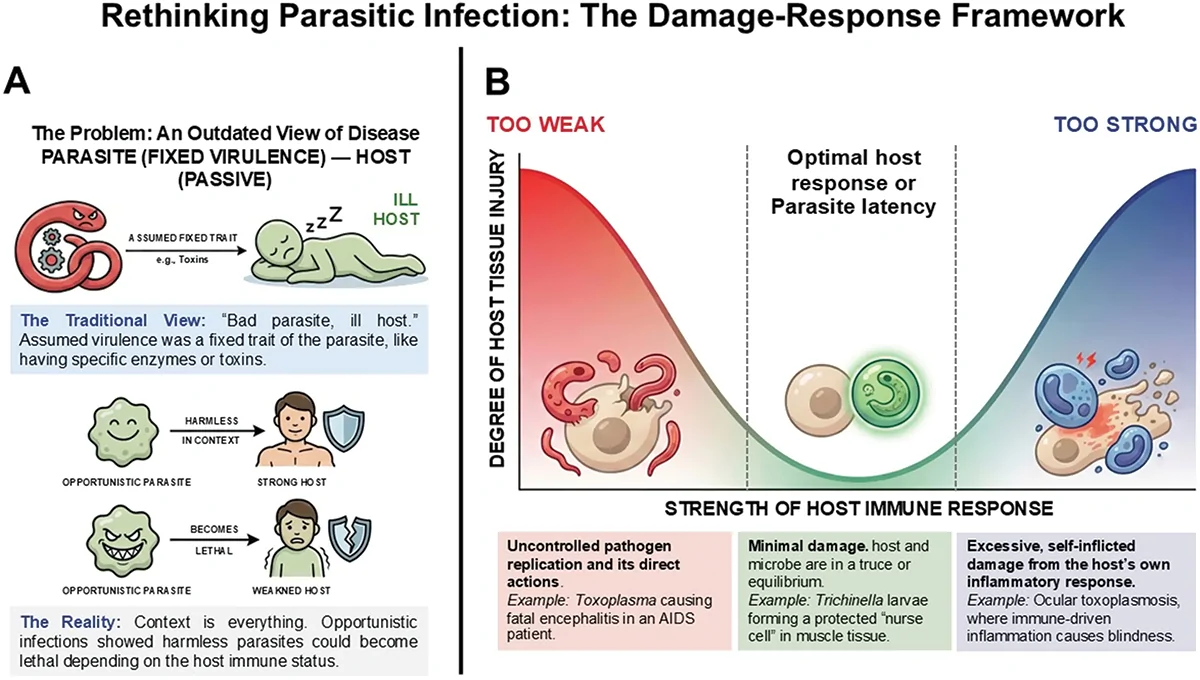
The DRF parabolic model of ParasiteInfection. (A) the traditional view (fixed virulence and disease outcome) of parasitic infections (B) the graphical representation of the DRF visualizes microbial pathogenesis as an emergent property of the host-microbe interaction, plotting the degree of host tissue injury (vertical axis) as a function of the strength of the host immune response (horizontal axis). The parabolic curve is key, illustrating that host damage is minimal at an optimal, intermediate level of immune activity, but increases at both extremes of the response spectrum. The figure highlights that disease outcome is not an intrinsic property of the microbe but results from an imbalance in the host-pathogen system.

From this basic parabola, Pirofski and Casadevall derived six standard categories of host-microbe interactions, each represented by a distinct curve shape [70]. The conceptual key of the DRF is visualized through a simple graphical representation of a parabolic curve that plots the degree of host damage (vertical axis) as a function of the strength of the host combat (horizontal axis). The prototypical parabolic shape of the curve is its most insightful feature (Figure 2). It illustrates that host damage is minimal at an optimal, intermediate level of immune activity, where the pathogen is controlled without overexuberant inflammation. However, damage increases at both extremes of the response spectrum:

A weak or insufficient response (the left side of the parabolic curve) fails to control microbial proliferation, leading to damage caused directly by the pathogen multiplication and various virulence factors.

An overly strong or exuberant host response (the right side of the parabolic curve) leads to excessive inflammation and immunopathology, where the host’s own combat mechanisms cause significant incidental tissue damage.

From this basic parabola, Pirofski and Casadevall derived six standard categories of host-microbe interactions, each represented by a distinct curve shape [70]. This provides a novel, functional classification system for pathogens based on the dynamics of their interaction with the host, rather than on their taxonomy or presumed virulence factors.

The DRF is originated in addressing clinical puzzles, such as the emergence of opportunistic infections during the HIV epidemic, revealing its nature as a top-down theory. Unlike models derived solely from controlled laboratory experiments, the DRF was designed to explain the vast heterogeneity of disease outcomes observed in human populations. This makes it an exceptionally powerful conceptual tool for physician-scientists, who must constantly bridge the gap between clinical observation and fundamental research. Nevertheless, the framework should be interpreted alongside additional biological determinants that are particularly relevant in parasitic diseases, including tissue-specific immune microenvironments, immune regulatory networks, the temporal evolution of chronic infection, parasite persistence strategies, metabolic adaptation of both host and parasite, and tissue remodeling processes such as fibrosis. These factors influence the magnitude, location, duration, and consequences of host damage and therefore complement, rather than contradict, the principles of the DRF. Likewise, parasite persistence should not be viewed simply as a state of immunological equilibrium; rather, it often represents a dynamic process of host–parasite accommodation in which immune regulation, partial parasite control, and ongoing tissue adaptation continuously evolve over time. Consequently, the framework necessitates a redefinition of core terminology. A “pathogen” is no longer an organism with a fixed arsenal of virulence factors but is defined as any microbe with the potential to cause damage in a susceptible host. Similarly, “virulence” is not an absolute measure but the relative capacity of a microbial pathogen to cause damage in a specific host context. This conceptual shift is substantial, reframing virulence as an emergent property of the host–microbe system rather than an intrinsic attribute of the microbe.

Importantly, the parabolic curve should be interpreted as a conceptual illustration of one central dimension of the DRF, namely the relationship between host damage and the overall effectiveness of the host response, rather than as a comprehensive mechanistic model encompassing every determinant of disease severity. The framework does not imply that inflammatory intensity alone dictates clinical outcomes, nor does it suggest that all host–parasite interactions can be reduced to a single continuum of immune activation. Instead, it serves as an overarching conceptual scaffold for understanding how damage emerges from host–microbe interactions. It should be emphasized that the six interaction classes of the Pirofski-Casadevall DRF are conceptual categories and are not intended to imply that every class is represented by a parasitic disease. Rather, the framework provides a pathogenic spectrum describing how host damage varies according to the balance between parasite virulence and host immune responses. Because parasitic infections are highly heterogeneous and dynamic, many lesions or manifestations do not fit exclusively into a single class and may shift along the spectrum depending on parasite burden, host immune status, stage of infection, and immune regulatory mechanisms. Therefore, the parasitic diseases discussed here are intended as representative examples illustrating the applicability of the DRF rather than exhaustive examples for all six interaction classes.

## The spectrum of parasite survival links to pathogenic trajectories and escape strategies in host pathogenesis

Interactions between parasites and their hosts may follow diverse trajectories, each dictated by parasite replication dynamics and the nature of the host immune response. These trajectories determine the course of pathogenesis, clinical manifestations, and long-term outcomes for both organisms. Because parasites comprise highly diverse taxonomic groups with distinct life cycles and immunological characteristics, the trajectories described below should be regarded as conceptual patterns rather than universal features shared by all parasitic infections. The relative contribution of parasite-derived virulence mechanisms and host immune responses varies substantially according to parasite species, tissue tropism, and host immune status. The following discussion outlines several illustrative examples.

### Acute symptomatic infection

This trajectory is characterized by rapid parasite proliferation, most prominently observed in unicellular organisms such as protozoa. The resulting infection elicits a vigorous and typically symptomatic immune response. Outcomes are often decisive; the host may successfully eliminate the parasite, or severe tissue damage. Intestinal protozoal infections such as giardiasis exemplify this pattern, commonly causing self-limiting acute diarrhea [71]. Although natural IgM contributes importantly to the early containment of infection and preservation of tissue homeostasis, the progression toward acute symptomatic disease is primarily determined by the combined effects of parasite burden, innate inflammatory activation, and the subsequent development of adaptive immune responses rather than by natural IgM activity alone. In this context, the response mediated by natural IgM plays a critical role in maintaining tissue homeostasis by promoting the clearance of apoptotic cells and damage-associated cellular debris. This immunoglobulin is produced constitutively, independently of prior antigen exposure, and exhibits polyreactivities that enable it to recognize neo-epitopes on damaged host cells such as oxidized phospholipids and subsequently initiate complement activation and phagocytic uptake to facilitate their removal. This process prevents cellular remnants from eliciting unwarranted inflammation and attenuates danger-associated signals arising from the innate immune system. Rather than representing a purposeful benefit mechanism, these immunoregulatory processes reduce excessive host-derived tissue injury while simultaneously creating conditions that may favor parasite persistence, depending on the biological characteristics of the infecting parasite. Such host-protective immunoregulatory activity corresponds to mitigating the severe pathological activities that manifest along the right side of the curve in the DRF [72,73].

### Chronic–persistent infection

In this scenario, the host immune response fails to achieve sterile clearance, yet succeeds in restraining parasite expansion. Parasites remain viable and continue to replicate, generally at low levels, driving sustained low-grade inflammation and cumulative tissue damage over years. However, chronic pathology extends beyond persistent low-grade inflammation and encompasses a broad spectrum of processes, including granulomatous inflammation, progressive fibrosis, vascular remodeling, repeated cycles of tissue injury and repair, and, in some infections, immune exhaustion or regulatory immune responses. The relative contribution of these mechanisms differs substantially among parasite species and affected organs.

Granulomatous lesion formation is commonly observed. This pattern typifies the indeterminate phase of Chagas disease and chronic schistosomiasis [74,75]. In chronic schistosomiasis, granulomas arise primarily in response to the deposition of schistosome eggs in tissues. Soluble egg antigens provoke a strong T_H_2-driven CD4 T-cell response, leading to cytokine production (e.g. IL-4, IL-5, IL-13) and the recruitment of effector cells that assemble around the eggs. Initially, granulomas served protective roles by sequestering toxic egg products and aiding egg excretion through host tissues. As infection persists, however, granuloma formation represents a dynamic process in which protective immune containment gradually transitions into pathological tissue remodeling. However, repeated cycles of granuloma formation and resolution ultimately result in progressive fibrosis, which underlies the major clinical complications of chronic disease such as portal hypertension and hepatosplenomegaly [33,76].

### Chronic–latent infection

This trajectory reflects a dynamic state of host–parasite interaction rather than a static equilibrium. During latency, parasite activity, immune surveillance, tissue repair, and immunoregulatory mechanisms continuously influence one another, producing a transiently stable condition that may shift in response to changes in host or parasite biology. After an initial acute phase, parasites transition into a quiescent or dormant state within host tissues, undergoing minimal or no replication. Concurrently, ongoing immune surveillance prevents reactivation. The resulting low-level interactions produce subclinical damage that remains below the threshold for overt disease. A prototype of latency is the differentiation of *T. gondii* into slowly dividing bradyzoites encysted within tissues; this serves as a long-term survival strategy in immunocompetent hosts, with the potential for reactivation under immunosuppression [77].

A transition toward the host perspective, host factors are equally crucial in determining the outcome of host–parasite interactions. The most influential variable is the status of the host immune system. Immunocompetent hosts may successfully contain *T. gondii* infection and maintain a benign latent state, whereas immunocompromised individuals, such as AIDS patients, may lose this control, leading to parasite reactivation and life-threatening encephalitis. Moreover, host genetic background may predispose individuals to specific immune response profiles (e.g. T_H_1 vs T_H_2 bias), thereby influencing susceptibility and clinical outcome, as demonstrated in leishmaniasis.

Co-infections with other pathogens can further reshape the immune landscape, either exacerbating or mitigating parasitic disease. Importantly, these survival trajectories should not be viewed as rigid, discrete categories but rather as positions along a dynamic spectrum. The DRF offers an effective graphical model to conceptualize this. A host position along the damage response parabolic curve tends to fluctuate and can shift over time due to changes in parasite activity, host immune status, or therapeutic intervention. For example, individuals with Chagas disease or American trypanosomiasis may transition from an acute, high-damage phase to a prolonged asymptomatic, low-damage chronic phase, and decades later progress to symptomatic cardiomyopathy characterized by renewed high damage as chronic inflammation exerts cumulative effects [78]. Similarly, latency is not a passive state, but rather an active, dynamic co-regulated host–parasite interaction requiring ongoing immune surveillance to prevent reactivation and continuous parasite-driven mechanisms to maintain quiescence. Rather than representing a permanent or stable equilibrium, latency reflects a continuously regulated biological state that may transition toward reactivation or sustained persistence depending on alterations in immune competence, parasite biology, or other biological factors. This dynamic interaction requires substantial metabolic investment, yet yields advantages for both sides, parasites secure a stable, long-term niche that supports future transmission, while hosts are spared the intense pathology that would accompany an unchecked, fulminant infection. Table 1 summarizes representative human parasitic diseases, their predominant positions on the DRF, and their associated immunopathological features.

**Table 1.** Representative human parasitic diseases mapped onto the DRF.

Parasite (disease)	Predominant position on the DRF continuum	Major mechanism of host damage	Dominant immune response	Representative clinical outcome
*Leishmania donovani* (Visceral leishmaniasis)	Left side (insufficient immune response)	Uncontrolled intracellular parasite replication	Impaired T_H_1/IFN-γ response	Progressive visceral disease
*Plasmodium falciparum* (Severe malaria)	Intermediate to right side (mixed parasite burden and excessive inflammatory response)	High parasite burden together with cytokine-mediated immunopathology	Excessive T_H_1/pro-inflammatory response	Cerebral malaria, severe anemia
*Entamoeba histolytica* (Amoebiasis)	Intermediate to right side (combined parasite invasion and inflammatory damage)	Direct tissue invasion plus host inflammatory injury	Innate inflammation with adaptive responses	Amoebic colitis, liver abscess
*Schistosoma mansoni* (Schistosomiasis)	Right side (excessive immune response)	Parasite egg-induced granulomatous inflammation and fibrosis	T_H_2-dominant granulomatous response	Hepatic fibrosis, portal hypertension
*Toxoplasma gondii* (Toxoplasmosis)	Left side in immunocompromised hosts; near the nadir of the curve in immunocompetent hosts	Opportunistic parasite proliferation following impaired immune control	T_H_1/IFN-γ dependent	Latent infection or disseminated toxoplasmosis

Note: The positions demonstrated represent the predominant location of each parasitic disease on the DRF curve under typical clinical conditions. Because host damage is dynamic, individual infections may shift along the continuum according to parasite burden, host immune status, genetic background, treatment, and other biological factors.

## Integrating the DRF: lessons learned from medically important parasitic infections

To illustrate the practical utility of the DRF, blastocystosis, toxoplasmosis, and trichinosis (trichinellosis) are discussed as representative mechanistic case studies. Rather than serving merely as descriptive examples, these diseases demonstrate how differences in parasite biology, host immune responses, and temporal changes during infection collectively determine the dominant source of host damage. Consequently, the DRF provides a conceptual framework for distinguishing parasite-driven injury from immune-mediated pathology, identifying disease stage-specific mechanisms, and informing the rational selection of therapeutic strategies. At the same time, these examples highlight important limitations of the framework, including the dynamic nature of host damage and the current lack of validated biomarkers capable of accurately positioning individual patients along the DRF.

### Blastocystosis through the lens of the damage–response framework

For over a century, the field of infectious diseases has historically been governed by the deterministic framework established by Robert Koch and Friedrich Loeffler, operating within a binary classification system that rigidly separates microorganisms into pathogens or non-pathogens. While this framework proved effective for identifying unequivocal pathogens such as *Mycobacterium tuberculosis*, it has begun to show its limitations as our understanding of the human microbiome has deepened. These limitations were brought into sharp relief by the discovery of organisms that defied conventional biological expectations, exemplified by *Blastocystis hominis* (now referred to taxonomically as *Blastocystis* spp.), the most prevalent eukaryotic parasite colonizing the human gastrointestinal tract. *Blastocystis* presents a perplexing clinical profile, in some hosts, it is associated with severe gastrointestinal disturbances, irritable bowel syndrome (IBS), and cutaneous hypersensitivity [79], whereas in the majority of carriers it behaves as a harmless commensal or even a mutualist linked to increased microbiome diversity and metabolic health [80–82]. This uncertainty implies that the very question of whether *Blastocystis* qualifies as a pathogen rests on a questionable premise. To resolve this inquiry, we must pivot to the DRF, which re-conceptualizes microbial pathogenesis as an emergent property of the dynamic interaction between host and microbe, where the sole relevant metric is the degree of host damage. Accordingly, this report examines the survival strategies and pathogenic potential of *Blastocystis* through the lens of the DRF, demonstrating that *Blastocystis* functions as a colonizing opportunistic pathogen (COP), whose nonlinear interactions with host immunity produce a parabolic damage curve [83]. This curve encompasses pathology at both extremes, immunodeficiency and hyper-immune reactivity while allowing for a stable, mutualistic state in immunocompetent hosts.

From a theoretical perspective, the DRF posits that pathogenesis arises from the interaction of host and microbe, and that host damage may originate from two distinct sources: microbial factors, such as enzyme and toxin production or tissue invasion, and host-derived factors, such as excessive inflammation resulting from attempts to eliminate the microorganism. For *Blastocystis*, this distinction is crucial. Although the organism possesses virulence factors capable of directly degrading host tissues, the most common clinical manifestations such as abdominal pain, diarrhea, and urticaria often reflect host inflammatory and allergic responses. This interaction can be conceptualized as a parabolic curve, with *Blastocystis* functioning as a class 4 pathogen that induces damage at both ends of the curve: unchecked proliferation in immunocompromised hosts, and immunopathology in hypersensitive hosts such as individuals with IBS. The midpoint of the curve the “nadir” represents asymptomatic colonization, where a balanced host response allows the organism to persist without causing disease. This aligns with the broader concept of COPs, which are capable of persisting asymptomatically in the microbiota for extended periods but can transition to pathogenicity when host variables shift. Biological and taxonomic re-consideration is essential for understanding *Blastocystis*. The organism is now classified within the phylum Stramenopiles, a group that is evolutionarily and biochemically distinct from classical medically important protozoa [84]. It possesses mitochondrion-like organelles (MLOs) adapted for cellular respiration. These MLOs exhibit metabolic flexibility, functioning in both aerobic and anaerobic environments, and can modify host immune responses while producing enzymes that potentially break down host IgA [81,85]. *Blastocystis* also exhibits substantial morphological plasticity, particularly its amoeboid form, which is often observed in symptomatic diarrhea and may indicate increased pathogenicity [86,87].

At the molecular level, damage is driven by key virulence factors, particularly cysteine proteases secreted to degrade host substrates and modulate host responses. In subtype ST7, legumain activates cathepsin B, which then disrupts epithelial tight junction proteins through the Rho-associated kinase (ROCK) pathway, leading to increased intestinal permeability. Proteases from ST4 and ST7 also activate NF-κB signaling in epithelial cells, inducing pro-inflammatory cytokines such as IL-8 and TNF-α, thereby recruiting leukocytes and driving tissue damage. Additionally, *Blastocystis* proteases can degrade secretory IgA, enabling the organism to evade mucosal trapping and weakening frontline host defenses [85,88–90]. Host-driven misdirecting factors within the DRF help account for the broad variability in disease manifestations. The microbiome acts as a “third party,” with eubiosis allowing resident bacteria to control *Blastocystis* populations, while dysbiosis facilitates overgrowth and can lead to marked pathogenicity. Host immunity determines where one falls along the parabolic curve. Individuals with impaired immunity, such as those with HIV/AIDS, suffer damage because the organism proliferates unchecked, while individuals with overly reactive immunity, such as those with IBS, experience damage driven by abnormal inflammation even when organism levels are low.

Synthesizing these lines of evidence yields a clear parabolic damage curve for *Blastocystis*. The left arm reflects immunodeficiency, where microbial factors drive epithelial destruction and severe diarrhea. The center of curve represents asymptomatic carriage, sustained by balanced immunity and a healthy microbiome. The right arm reflects immunopathology in conditions such as IBS or chronic urticaria, where host-derived cytokine release produces pain and cutaneous symptoms [91]. Overall, the DRF portrays *Blastocystis* spp. as a shape-shifting COPs whose virulence traits are insufficient to predict pathology without considering host context. From a clinical perspective, the DRF suggests that management should not rely solely on the detection of Blastocystis but should incorporate host-related factors, including immune status, gastrointestinal inflammation, and microbiome composition. This perspective may explain why eradication therapy benefits only selected patients despite widespread asymptomatic colonization. Nevertheless, reliable biomarkers capable of distinguishing parasite-driven disease from host-mediated pathology remain unavailable, limiting the direct clinical implementation of this conceptual framework.

### Toxoplasmosis through the lens of the damage–response framework

*T. gondii*, an obligate intracellular apicomplexan (sporozoan) protozoan, serves as perhaps the most sophisticated biological model for this framework [92]. Capable of infecting virtually all warm-blooded animals and persisting in one-third of the human population, *T. gondii* navigates the entire breadth of the damage-response curve [93]. It is a pathogen that can kill through uncontrolled replication (the left side of the DRF curve), persist benignly as a latent cyst (the trough of the curve), or blind the host through hyper-inflammatory uveitis (the right side of the curve). This report provides an analysis of *T. gondii* through the lens of the DRF, integrating parasitology, immunology, and evolutionary biology to deconstruct how this parasite manipulates the host-pathogen interface. For *T. gondii*, the curve is classically parabolic. On the “left side” (weak response), such as in AIDS or fetal immaturity, the parasite replicates uncontrollably, and damage is driven by microbial factors like lysis of host cells. The “trough” of the curve, representing the optimal response, denotes a balanced, intermediate immune state (T_H_1- biased) in which the host restricts parasite replication while avoiding self-inflicted tissue injury, resulting in latency or low-grade chronic infection with negligible tissue alteration. Finally, the “right side” (excessive response) occurs when the immune response becomes unregulated or hyper-intense (e.g. ocular toxoplasmosis, IRIS). In this state, the host immune system destroys tissues in an attempt to clear the parasite, leading to immunopathology [93,94]. Under the DRF, *T. gondii* is best described as a class 3 pathogen, depending on the specific host-strain combination, as it causes damage at both extremes of the immune response. This classification dictates therapeutic strategy, under class 3 pathogens, as the selected treatment must vary based on the patient’s position on the curve. Immunodeficient patients require immune boosting or antimicrobial agents (moving right toward the trough), while hyper-inflammatory patients require immunosuppression (moving left toward the trough).

The pathogenesis of *T. gondii* is inextricably linked to its ability to switch between two life-cycle stages, each interacting differently with the host immune combat [95]. Tachyzoites are the rapidly dividing, motile form responsible for acute dissemination. They employ a lytic cycle involving gliding motility powered by an actomyosin motor, active invasion to create a specialized parasitophorous vacuole (PV) that does not fuse with lysosomes, replication by endodyogeny, and, finally, egress and lysis of the host cell [96,97]. Conversely, bradyzoites are the agents of persistence, residing within intracellular tissue cysts. The cyst wall acts as a cloak, protecting the parasite from immune surveillance and allowing it to persist for the lifetime of the host. Importantly, bradyzoites are resistant to low pH and digestive enzymes like pepsin, an adaptation that ensures survival during passage through the stomach of the definitive hosts. *T. gondii* actively rearranges the host cell environment using effectors secreted from apical complex organelles. Inside the apical complex, micronemes mediate attachment, while rhoptries are secreted during invasion to intercept acute immune signals [98]. The “left side” of the DRF parabola represents the scenario where the host immune response is insufficient to control parasite replication. In this context, damage is directly proportional to the parasite burden. When immunosurveillance fails, the lytic cycle of the tachyzoite proceeds unchecked, causing mechanical and cytotoxic damage. The exponential replication of tachyzoite consumes host resources and physically ruptures host cells, leading to multifocal necrosis in vital organs like the liver, lungs, and brain [99]. To maximize replication time, *T. gondii* actively inhibits host cell apoptosis by blocking caspase-3 and caspase-8 activation [100]. In the absence of an adaptive immune response, this results in a higher parasite load per cell and eventually massive, synchronized lysis of tissues [93].

The clinical relevance of the left arm is most dramatic in patients with defects in T cell immunity, such as those with HIV/AIDS or transplant recipients. In patients with CD4^+^ T cell counts < 100 cells/µL, the immune pressure that maintains bradyzoite cysts is lost, allowing reactivation where bradyzoites differentiate back into tachyzoites [101,102]. The resulting uncontrolled replication in the brain leads to necrotizing abscesses. Similarly, solid organ transplant recipients are at risk from reactivation or primary infection via a mismatched organ, often leading to disseminated disease. The nadir of the DRF parabola represents the ideal evolutionary outcome for an intermediate host: survival with chronic infection. This state, termed latency or dormant stage, acts as a truce where the host limits damage and the parasite secures a reservoir for transmission. This equilibrium is maintained by a robust type 1 immune response. Cytokine IL-12 drives the production of IFN-γ, which restricts parasite growth and activates intracellular killing mechanisms. Both CD4^+^ and CD8^+^ T cells are required to maintain the cyst state, depletion of either leads to reactivation [103].

Rather than representing a simple dormant state, latency constitutes an active developmental pathway regulated by how the parasite senses and responds to host stress. Recent research has identified BFD1 as a Myb-like transcription factor that plays a critical role in the chronic differentiation of *T. gondii*, serving as the master transcriptional regulator of this process. Host immune pressure, nutrient deprivation, or pH changes trigger the parasite stress response, leading to the phosphorylation of eukaryotic IF2α [104,105]. This suppresses general protein synthesis but enhances the translation of BFD1 mRNA, which in turn drives the expression of bradyzoite-specific genes [106,107]. The tissue cyst itself is a dynamic structure requiring active maintenance. Cysts show an inclination for immunological privileged sites, particularly the brain and muscle, where they are shielded from direct cytotoxic attack. Even in the latent state, the presence of cysts provokes a low-level, sterile inflammatory response, which acts as a surveillance mechanism to kill any spontaneously reactivating bradyzoites, preventing a return to the “left side” of acute disease. The “right side” of the DRF parabola describes scenarios where the host immune response is too vigorous, resulting in damage that exceeds that caused by the parasite itself. This is the domain of immunopathology.

A classic model is the lethal ileitis observed in C57BL/6 mice following oral infection, where an uncontrolled “hyper inflammatory cytokine response” of T_H_1 cytokines (IFN-γ and TNF-α) causes necrosis of the gut mucosa. Treatment with neutralizing antibodies against these cytokines prevents death, confirming that the immune response is the agent of mortality [108]. In human clinical aspects, the immune paradoxical reaction in HIV patients serves as a prime example. The initiation of antiretroviral therapy (ART) leads to a rapid recovery of CD4^+^ T cells, which may recognize residual *Toxoplasma* antigens and mount an exaggerated inflammation. This results in severe cerebral edema and worsening neurological symptoms despite controlled parasite numbers [109,110]. Similarly, ocular toxoplasmosis often involves a type IV hypersensitivity reaction to parasite antigens released upon cyst rupture, causing vision-threatening damage [111]. The standard treatment involving both antimicrobial agents (to control parasite replication) and corticosteroids (to suppress host inflammation) implicitly aligns the DRF by attempting to manage both sides of the damage curve [112].

Recent studies have shown that the effector TgIST translocates to the host nucleus, binds phosphorylated STAT1, and recruits the NuRD repressor complex to deacetylate histones at STAT1-target promoters. This renders the cell refractory to IFN-γ signaling, blinding it to immune activation and shifting the interaction toward the “left side” [113–115]. The parasite also fine-tunes inflammation using ROP16 and GRA15. ROP16 induces an anti-inflammatory M2 alternative macrophage to avoid right side hypercytokinemia [116], whereas GRA15 activates NF-κB to drive inflammation, explaining why type II strains (variants) are more often associated with inflammatory ocular disease than type I and type III strains [117,118].

Overall, these findings demonstrate that the position of an infected individual along the DRF is determined by continuously changing interactions between parasite developmental stage, parasite-derived effectors, and host immune responses. Rather than representing a fixed characteristic of either the parasite or the host, disease severity emerges from these context-dependent biological processes. Clinically, this concept provides a rationale for combining anti-parasitic therapy with immunomodulatory treatment in selected situations such as ocular toxoplasmosis or immune reconstitution inflammatory syndrome (IRIS). However, translating this framework into routine practice will require biomarkers capable of quantifying both parasite activity and host inflammatory responses. Vaccinology and biologic therapies must also navigate the DRF, aiming to induce a T_H_1 response strong enough to prevent acute disease but regulated enough to avoid severe immunopathology [119].

### Trichinosis through the lens of the damage–response framework

Infection with the tissue nematode *Trichinella spiralis* offers an exceptional model for understanding long-term parasite survival within a host. It illustrates a suite of highly specialized strategies involving both the activation and the active modulation of host immune responses to establish one of the most enduring chronic infections known. The infection proceeds through two distinct phases: an initial enteral (intestinal) phase and a subsequent parenteral (invasive) phase, during which larvae become embedded within skeletal muscle fibers. Beyond its value as an experimental model of host–parasite interactions, *T. spiralis* is also an important One Health pathogen because its transmission is intrinsically linked to the interconnected health of humans, domestic animals, wildlife, and the environment. The parasite circulates through both domestic and sylvatic transmission cycles, with infection occurring primarily through the consumption of raw or undercooked meat containing infective larvae [120,121]. Each phase aligns with different landscapes of the DRF curve [122]. Following the ingestion of contaminated meat, *T. spiralis* larvae mature into adult worms within the small intestine, producing gastrointestinal symptoms. Female worms then release newborn larvae (NBL), which disseminate hematogenously to skeletal muscle [123]. This migratory phase elicits an early T_H_1-biased immune response that drives intense inflammation and results in the classic manifestations of trichinosis such as myositis, fever, periorbital edema, and acute urticaria. During this stage, the host occupies the right side of the DRF curve, where the pathology is predominantly immunopathological arising largely from the host inflammatory response to migrating larvae [124,125]. Based on the DRF, *T. spiralis* is a class 3 pathogen because host damage is maximal when the immune response is either too weak (disseminated infection) or too strong (severe inflammation). The parasite survival strategy is to force the host into the intermediate, low-damage zone.

*T. spiralis* does not merely withstand this inflammatory assault, it actively reshapes the host immune system. Clear examples include the parasite use of ES products, such as thioredoxin peroxidase 2 (TsTPX2) to suppress T_H_1 responses and promote a T_H_2-dominant milieu that is essential for parasite survival [126], [127]. Furthermore, a study by Sun et al. (2019) demonstrated that both adult worm ES products (AES) and muscle-larva ES products (MES) contain diverse immunomodulatory molecules that redirect host immunity from a destructive T_H_1 profile toward a T_H_2 and regulatory T-cell (T_Reg_)–enriched response [128]. This T_H_2 response, characterized by cytokines such as IL-4 and IL-10, is inefficient at parasite clearance but promotes tissue repair and dampens inflammation. This deliberate immunomodulation shifts the host leftward along the DRF curve away from the peak of immunopathological damage and toward a state of reduced host injury.

A histological hallmark of *T. spiralis* infection is the formation of the nurse cell. The larva invades a single skeletal muscle cell and induces a dramatic transformation, generating a capsule-like structure that both protects the parasite and supplies nutrients. This cellular niche enables the larva to persist for months or even years with minimal ongoing host damage [129]. Importantly, the nurse cell is not a parasite-derived cyst – although sometimes referred to as “cystogenesis” – but a structure composed entirely of host elements [129,130]. The process resembles muscle regeneration, the infected cell dedifferentiates, reenters the cell cycle, and arrests at the G2/M transition. Concurrently, satellite cells proliferate, differentiate, and fuse with the infected fiber, replacing much of its cytoplasm [131]. Moreover, ES products trigger the host TGF-β/Smad3 signaling pathway, driving a massive upregulation of collagen types IV and VI which forms a thick fibrous capsule around the nurse cell [132], [133]. The parasite also induces robust angiogenesis around the capsule to meet its long-term metabolic requirements [134], [135]. In the aspect of immunological tolerance, nurse cells thus become an immunologically privileged site, enabling a highly immunogenic parasite to survive in an immunocompetent host. This immune privilege arises not only from anatomical compartmentalization but also from the continuous cellular cross-talking. The ES products continuously shape the local immune environment by inducing tolerogenic dendritic cells, which promote T_Reg_ differentiation. This skews the local response toward a T_H_2/T_Reg_ profile elevating anti-inflammatory cytokines such as IL-10 and TGF-β while suppressing inflammatory effector mechanisms [136–139].

**Figure 3.**
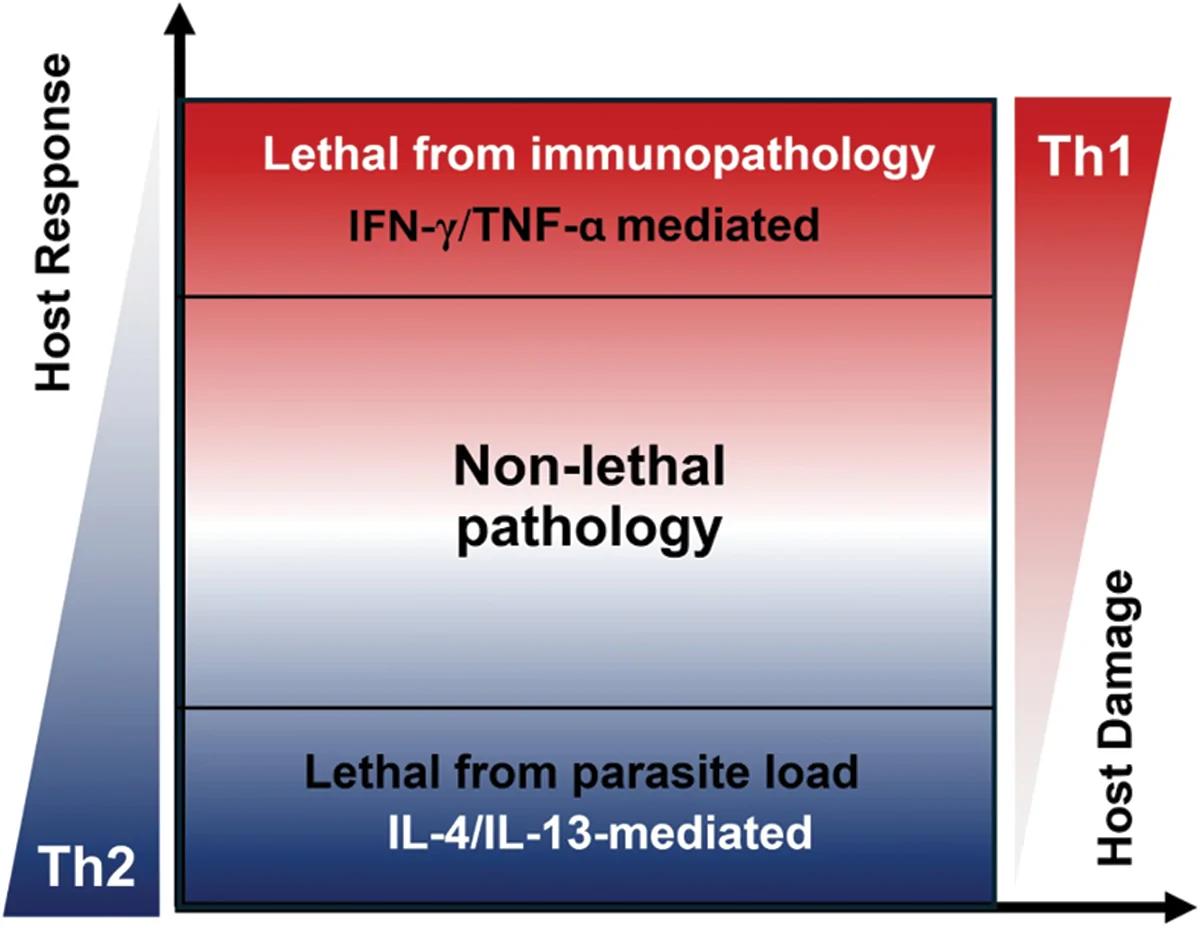
Parasites can modulate the host T cell immunity in highly complex ways through sophisticated immunomodulatory strategies. Because parasites rely on hosts throughout their life cycles, their long-term survival and transmission depend on maintaining host viability across generations. Their primary aim is maintaining an immunological balance that protects both parasite and host from significant damage. The DRF emphasizes that clinical outcomes arise from the dynamic interaction between host and pathogen. Successful parasites manage this interaction to keep the host near an equilibrium point, often corresponding to a latent state where host damage remains minimal. This balance is especially important in definitive and intermediate hosts, where the parasite must complete its developmental cycle. A second principle involves reprogramming host immunity. Immune modulation is a central strategy that limits harmful inflammation and promotes chronic persistence by redirecting the immune response. Many parasites promote a shift toward T_H_2 and T_Reg_ responses. For example, *T. spiralis* secretes immunomodulators (es products) that redirect the host response from an inhibitory T_H_1 profile toward a T_H_2 or T_Reg_ profile that supports host tissue repair. This redirection enables the parasite to induce “nurse cell” formation, allowing long-term survival in host muscle. T_H_2 responses play an important supportive role in this process. However, prolonged T_H_2 polarization may drive fibrosis during chronic infection, ultimately causing loss of organ function and severe disease. Even so, this redirection generally protects the host from extreme immunopathology driven by overly strong T_H_1 responses mediated by IFN-γ and TNF-α. In conclusion, the ability of parasites to guide the direction of host immunity plays a decisive role in determining whether infection remains asymptomatic or progresses to severe disease. This modulation can move the host toward either side of the parabolic curve (modified in part from Wynn & Kwiatkowski, [45]).

The trichinosis model illustrates how sequential changes in parasite biology and host immune responses alter the predominant mechanism of tissue injury throughout infection. Consequently, the DRF emphasizes that therapeutic approaches should consider disease stage, balancing anti-parasitic treatment during active larval migration with strategies aimed at limiting excessive inflammatory damage when immunopathology predominates. Although this interpretation remains largely conceptual, it provides a useful framework for future studies seeking biomarkers that distinguish parasite-mediated injury from immune-mediated tissue damage (Figure 3).

Although the DRF provides an integrative framework for understanding parasite pathogenesis, several limitations should be acknowledged. First, the relative contribution of parasite-mediated injury and immune-mediated pathology is highly dynamic and frequently changes during the course of infection. Second, different organs within the same patient may occupy different positions along the damage–response dynamic simultaneously. Third, quantitative biomarkers capable of objectively determining an individual’s position on the DRF curve remain unavailable for most parasitic diseases. Finally, parasite genetic diversity, host genetic variation, co-infections, nutritional status, and microbiome composition introduce additional biological complexity that cannot be fully represented by a single conceptual curve. Consequently, the DRF should be regarded as a heuristic framework that guides mechanistic interpretation rather than as a predictive clinical model. Future studies integrating systems immunology, quantitative biomarkers, and longitudinal patient data will be essential for translating this framework into precision medicine.

## Conclusion and future perspectives

This review emphasizes the importance of understanding the DRF within a broader perspective that captures the interactions and dynamic relationships between hosts and pathogens. Rather than serving as a predictive model of pathogen behavior or replacing existing genetic or evolutionary theories, the DRF provides an integrative conceptual framework for interpreting how variations in host immune responses and parasite biology collectively determine the extent of host damage. Genetic models, such as the gene-for-gene (GFG) paradigm and the matching allele (MA) system, operate at a distinct conceptual level and provide complementary insights into host–pathogen interactions [140,141]. The GFG and MA focus on molecular specificity by asking the question *“Can infection occur?”* They describe the outcome of recognition events between host resistance proteins and pathogen effector molecules. The DRF operates at a higher physiological level. It does not center on the initial recognition event but on the quantitative and qualitative outcomes that follow. It addresses a different question, *“Once infection is established, what determines the clinical outcome?”* The framework does not replace genetic models but complements them. Genetic interactions may determine whether a parasite can successfully initiate infection, but the DRF explains the full range of possible outcomes, from asymptomatic carriage to severe disease, which depend on the dynamic behavior of the host immune response. The framework also provides a revised view of the evolution of virulence. Traditional ideas such as the avirulence hypothesis, which predicts that parasites evolve toward reduced harm [142], and the trade-off hypothesis, which suggests that virulence is maintained as a necessary cost of transmission [143], treat virulence as a selectable property of the pathogen. In contrast, the DRF views virulence as a property that emerges from the interaction between host and parasite. Parasites do not possess a fixed level of virulence. They have the potential to cause damage, and that damage becomes apparent only within the specific biological context of the host.

From the perspective of the DRF, host–parasite interactions should therefore be regarded as dynamic biological processes rather than progression toward any predetermined or evolutionarily preferred state. Clinical outcomes continuously shift according to changes in parasite burden, parasite virulence, host immune responsiveness, host genetics, environmental influences, and therapeutic interventions. Consequently, persistent parasitic infections represent evolving positions along the host damage continuum rather than stable equilibria. This perspective suggests that natural selection may act not on virulence itself but on the ability of parasites to adjust host responses in ways that improve their own survival and transmission. Instead of implying that parasites evolve toward an “optimal” state, the DRF emphasizes that prolonged persistence, latency, immune-mediated pathology, or parasite clearance each represent context-dependent outcomes arising from specific host–parasite interactions. None of these outcomes should be interpreted as biologically predetermined or universally advantageous. This concept aligns with disease tolerance, in which hosts evolve mechanisms that reduce damage without necessarily eliminating the pathogen [144]. Within the DRF, this state corresponds to maintaining a low position on the curve associated with minimal damage even while the parasite remains present. The wide variety of survival patterns seen in medical parasitology, ranging from brief self-limiting infections to lifelong persistence, can be understood through this framework. Many nematodes and commensal intestinal protozoa can be positioned clearly along the characteristic parabolic curve described by the model. This visual representation underscores that the severity of the disease is governed by both the strength and the character of the host immune response.

Finally, the DRF offers a valuable conceptual foundation for future research aimed at elucidating the mechanisms that regulate host damage during parasitic infections. As our understanding of host immune heterogeneity and parasite biology continues to advance, this framework may facilitate hypothesis generation, biomarker discovery, the development of predictive models of disease severity based on integrated host and parasite determinants, and the design of host-directed therapeutic strategies intended to minimize immunopathology while preserving protective immunity [145]. Nevertheless, these potential translational applications remain disease-specific and require rigorous experimental, translational, and clinical validation before broad implementation can be justified. By emphasizing host damage as an emergent property of continuously evolving host–parasite interactions rather than an intrinsic characteristic of either organism alone, the DRF provides a robust conceptual framework for interpreting the remarkable biological diversity and clinical complexity of parasitic diseases.

## Data Availability

There is no data associated with this research.
